# The correlation between lifestyle health behaviors, coping style, and mental health during the COVID-19 pandemic among college students: Two rounds of a web-based study

**DOI:** 10.3389/fpubh.2022.1031560

**Published:** 2023-01-12

**Authors:** Yi Zhang, Shuman Tao, Yang Qu, Xingyue Mou, Hong Gan, Panfeng Zhou, Zhuoyan Zhu, Xiaoyan Wu, Fangbiao Tao

**Affiliations:** ^1^Department of Maternal, Child and Adolescent Health, School of Public Health, Anhui Medical University, Hefei, China; ^2^NHC Key Laboratory of Study on Abnormal Gametes and Reproductive Tract, Hefei, China; ^3^MOE Key Laboratory of Population Health Across Life Cycle, Hefei, China; ^4^Department of Nephrology, The Second Hospital of Anhui Medical University, Hefei, China

**Keywords:** depression, college students, COVID-19, lifestyle health behaviors, anxiety

## Abstract

**Background:**

During the last months of 2019, worldwide attention has focused on a pandemic of COVID-19, and the pandemic spread rapidly, becoming a public health emergency of international concern. The Chinese government has quickly taken quarantine measures and the drastic measures incurred to curtail it, which could have harmful consequences for everyone's behavior and mental health.

**Objective:**

This study aimed to investigate the correlation of influencing factors and mental health symptoms among Chinese college students according to two rounds of surveys during the COVID-19 pandemic.

**Materials and methods:**

This study was divided into two stages of cross-sectional investigation. In February 2020 and May 2020, two cross-sectional surveys were conducted on college students in the above areas by means of cluster sampling. From February 4 to February 12, 2020, 14,789 college students completed the first round of online study from 16 cities and universities in 21 in China, excluding participants who completed the questionnaire, and finally included 11,787 college students. After 3 months, we also conducted a large-scale questionnaire survey based on the first study area to observe the behavioral changes and psychological symptoms of college students within 3 months. Using the convenience sampling method, a survey on the cognitive and psychological status of COVID-19 among college students was conducted in 21 universities in 16 regions in May 2020. The regions among the two surveys are divided into Wuhan (District 1), neighboring Hubei (District 2), first-tier cities, namely, Beijing, Shanghai and Guangzhou (District 3), and other provinces (District 4).

**Results:**

(1) In the first survey, the average age of the participants was 20.51 ± 1.88 years. One-third of the college students were male. In total, 25.9 and 17.8% of college students reported depression and anxiety symptoms, respectively. College students, who reported higher ST, lower PA, higher soda beverage and tea beverage intake, have Chinese herbal medicine and vitamin drinking, and decreased frequency of diet had higher depression and anxiety symptoms. Moreover, infection risk, perceived resistance to COVID-19, concerns about physical symptoms, family/friends directly/indirectly contacted with confirmed cases, and seeking psychological counseling had significant associations with anxiety and depression symptoms. (2) In the second round of surveys, 6803 males (41.7%) and 9502 females (58.3%) had a mean age of 20.58 ± 1.81 years. The prevalence of depressive symptoms and anxiety symptoms during COVID-19 was 30.7 and 23.9%, respectively. (3) In both surveys, college students who reported having higher ST, lower PA, higher soda beverage and tea beverage intake, Chinese herbal medicine and vitamin drinking, and decreased frequency of diet also had higher depression and anxiety symptoms. Coping styles with COVID-19 partially mediate the association between some related lifestyle behaviors and anxiety and depression. The results of the conditional process model analysis support our hypothesis that lifestyle behaviors, and coping styles are both predictors of anxiety and depression symptoms, with both direct and indirect effects moderated by gender level. In logistic regression analysis, the correlation of depression and anxiety in the second rounds of the survey was higher than that in the first survey. In two surveys, there was a correlation between lifestyle health behaviors and coping style, gender and mental health.

**Conclusions:**

Our findings demonstrated that the prevalence of anxiety and depression in the two rounds of surveys was different, and the prevalence in the second round was higher than that in the first round, as well as that in the two rounds of surveys. All survey identified that unhealthy lifestyle behaviors were positively associated with depression and anxiety symptoms. Compared with the emerged city of risk areas, other pandemic regions have a lower risk of mental health problems. The survey findings complement each other. Lifestyle health behaviors and coping style alleviated mental health symptoms. COVID-19-related social stressors were positively associated with mental health symptoms. The study also suggests that at the beginning of the pandemic, the effective management of college students' mental health problems, corresponding coping measures and maintaining a good lifestyle play a key role in the prevention and control of other cities. Understanding the college students' lifestyle behaviors during COVID-19 lockdown will help public health authorities reshape future policies on their nutritional recommendations, in preparation for future pandemics.

## Introduction

During the last months of 2019, worldwide attention has focused on a pandemic of COVID-19, which has led to global instability (1–3). Soon after, based on previous experience–public health prevention strategies are likely to remain the measure of choice for disease prevention (4), governments around the world have implemented a variety of containment and mitigation strategies to adequately address the adverse impacts of COVID-19 across the country, asking people to stay at home and avoid going out and take protective measures to prevent the further spread of COVID-19 to the greatest extent (5, 6). Globally, as of 5:50 pm CEST, 12 July 2022, there have been 554,290,112 confirmed cases of COVID-19, including 6,351,801 deaths, reported to the WHO (7). The world is experiencing an extraordinary life-changing challenge as a result of the COVID-19 pandemic.

It is difficult to predict exactly when the COVID-19 pandemic will subside, so many places have changed their habits and become accustomed to a new normal— “social distancing” is now part of daily life (8). In prolonged home quarantine, especially at the beginning of the pandemic, because of “social distancing,” people's lifestyle behaviors may change greatly; at the same time, negative effects have also emerged, and people's mental health may also be greatly affected (9). According to previous study, home confinement and social distancing are two of the main public health measures to curb the spread of SARS-CoV-2, which can also have harmful consequences on people's mental health (9). Among them, the percentage of children reporting elevated emotional difficulties rose from 17 in 2019 to 27% in 2021 (10); in addition, the CO-SPACE study conducted in Oxford, UK, reported that childhood mental health deficits increased during the first lockdown, recovered to some extent, but increased during subsequent lockdown (11). A pooled analysis of 29 studies since the start of the pandemic estimates that one in four and one in five young people experience elevated symptoms of depression or anxiety, respectively, with estimates of psychological problems higher in studies conducted during the middle and later stages of the pandemic (12). In the United Kingdom, higher levels of anxiety (21–37%) were reported during the pandemic in 2020 compared with 2019 (13). Guessoum et al. review the literature on adolescent psychiatric disorders related to the COVID-19 pandemic and lockdown and indicated the COVID-19 pandemic and lockdown may have a negative impact on the mental health of adolescents, although there is still no data on the long-term impact of this crisis (5).

In addition to isolation and containment, there are also indirect factors that affect psychology. Data show that physical activities that promote physical and mental health have been largely disrupted during the pandemic as a result of isolation, while a significant reduction in activity space outside of isolation, coupled with the rise of online activity, has led to an increase in the use of screen media for education and entertainment (14). Previous studies have proven that long-term home quarantine leads to changes in daily activities, including physical activity (PA) (mainly indoor PA) and screen time (ST), and unhealthy lifestyle habits, such as the intake of health care products. However, the purpose of these actions is actually to find information about the spread and travel trajectories of confirmed cases, both for accurate and up-to-date information about the virus itself and for the protective measures taken in response to the pandemic to make informed decisions and improve health (15–17), in a sense, because of the uncertainty about the harm caused by COVID-19. For example, Schmitt et al. found that during the first COVID-19 lockdown in Germany, PA decreased while leisure ST increased (18). In addition, children reported an average (±SD) of 3.9 (±2.2) days per week with at least 60 min of physical activity and 4.4 (± 2.5) h/day of recreational ST (19). Together, these data suggest that PA levels have declined and ST has increased among children and adolescents since the COVID-19 pandemic (15, 17). In addition, other unhealthy behaviors such as sugar-sweetened beverages (SSBs) also suggested a positive association between the crude infection rate, the crude mortality rate and SSBs, while fruits showed negative effects on the crude infection rate and the crude mortality rate (20).

Due to the sudden and widespread spread of COVID-19, a lot of information overload (misinformation and false positives) in the media can have a huge impact on the communication of disasters and emergencies, which can lead to bad-tempered, anxiety, worry and depression (21, 22). In addition, it is also possible that changes in lifestyle behavior during home isolation may disrupt normal lifestyle rhythms, thereby increasing mental health problems (17, 23), which will have a profound impact on public sentiment and behavior during COVID-19, both directly and indirectly, such as changes in sleep and dietary rhythms and the intake of unhealthy diet behaviors including SSBs and healthcare products consumption (24). Moreover, Lesser et al. have explored that there was well-being differences and changes in PA within the active and inactive groups, the inactive participants that were more active or maintained their activity levels indicated higher levels of social, emotional and psychological health (25, 26). We also understand that regular sleep habits are crucial for our physical health, so maintaining regular diet rhythmicity and avoiding unnecessary disrupted rhythms regardless of sleep or diet are important for our health (27, 28). Behavioral changes among national college students have not been widely reported in previous studies during pandemic.

These clear results highlight the urgent need for improved prevention and may provide a basis for corresponding interventions. For a long time, lifestyle health behaviors have played an important role in the pathogenesis of chronic diseases, which provides a theoretical basis for behavioral and mental health recommendations for school-age children and adults. Moreover, positive coping styles were associated with reduced negative mental health symptoms. However, there are few studies on the overall behavior changes of college students nationwide during the COVID-19 pandemic and its impact on college students in different regions (29).

In the current study, only a few studies have explored behavioral changes and mental health among college students in the early days and within 3 months of the pandemic. Based on previous study design (28), as evidenced by two rounds of research, monitoring psychosocial problems and related influencing factors among adolescents from the COVID-19 pandemic is a public health priority that requires urgent and effective action at multiple levels of society. This study aims to partially further fill this gap. Therefore, our study **first** aimed to investigate the prevalence of health behaviors among college students from the beginning to 3 months after the COVID-19 pandemic. **Second**, we examined the associations between lifestyle, healthy behaviors, and mental health status. Specifically, we also assessed health behaviors and mental health in different school areas. **Finally**, most of the previous studies focused on the psychological and behavioral changes before and after the pandemic of the pandemic, while our study focused on the psychological and behavioral changes of college students at the beginning of the pandemic and 3 months after the pandemic. We also displayed the behavior difference between two rounds of survey to provide some suggestions. For these reasons, and given that pandemics can extend from weeks to months, two successive surveillance surveys provide a sound rationale for the development of appropriate interventions for healthy family lifestyle behaviors to reduce the negative psychological impact during pandemics.

## Materials and methods

### Setting and participants

This study is divided into **two stages** of cross-sectional investigation. In February 2020 and May 2020, two cross-sectional surveys were conducted on college students in the above areas by means of cluster sampling. This first nationwide cross-sectional online survey was described in previous study (30). A two-stage sampling strategy was used (30). Finally, after removing the participants without completed questionnaires, 11,787 (Supplementary Table S1) participants from 16 cities and 19 colleges in China were involved in the current study, and we followed the methods of Zhang et al. (30, 31). Referring to the region and university of the first survey, in May 2020, 21 universities were selected from 16 regions to conduct the second survey, and a total of 16,305 people completed the questionnaire. At the time of the second survey, the students are still under lockdown for several months.

### Procedure

They completed the questionnaires in Chinese college students through an online survey platform (“Survey Star [https://www.wjx.cn/]”). The Ethics Committee of Anhui Medical University approved this study (the number of ethical approvals is 20200319). All respondents provided informed consent. Data collection took place over several days (4 February to 12 February 2020 and May to June 2020).

### Survey variables

The study included some questions related to the COVID-19 pandemic from a two rounds of survey. The structured questionnaire consisted of questions that covered several aspects: (1) sociodemographic data; (2) whether someone had pneumonia in the past 14 days and tourism history in risk areas; (3) contact history with COVID-19 patients in the past 14 days; (4) knowledge and concerns about COVID-19; (5) precautionary measures against COVID-19 in the past 14 days; (6) additional information required concerning COVID-19; (7) lifestyle health behaviors with COVID-19 and coping style; and (8) mental health symptoms. Sociodemographic data were collected on gender, age, grade, student type, regional areas and school areas. Lifestyle health behaviors with COVID-19 were measured with the following fields: ST, PA, SSBs, frequency of diet, health food (including Chinese herbal medicine or vitamin), appetite and vigor change.

The ST of participants was measured with one question: “How much time do you spend on screen time?” (32). ST was categorized as > 4 h/d (high), 2–4 h/d (medium) and ≤ 2 h/d (low) (30, 33–35).

Participants' PA was measured using the question, “On how many days in the last week did you spend at least an hour (60 min) on PA?” (That is, the total amount of PA in a day makes your heart beat faster and sometimes your breathing significantly faster)?” The options range from 0 to 7 days (36) High PA was defined as at least three days per week (PA≥3 days) of exercise (30, 31). This item has previously been used in large student surveys (30), and has been shown to provide relatively comprehensive access to information on health behavior to generate effective and reliable responses (37).

The SSBs of participants were measured with two questions: during the last week, how many sugar sweetened beverages do you drink per day? The answers were: none, < 1 bottle per day, one bottle per day, two bottles per day, three to four bottles per day, and more than four bottles per day (30); The frequency of diet was measured with one question: during the last week, do you feel your sleep was more regular? The answers were increased, unchanged and decreased (30).

Health food was measured with two questions: during the last week, do you consume Chinese herbal medicine/vitamin? The answers were yes and no (30).

Appetite and vigor changes were measured with two questions: compared with before, if you feel worse appetite than before or worse vigor than before? The answers were none, sometimes, half of the day, all the time (30).

Copying style with COVID-19 questionnaire was measured by following questions: (1) Like to keep the unhappy things in mind, but never forget it; (2) When you encounter setbacks, you usually compare them with similar people to make sense; (3) In the face of painful events, can be used to alleviate the pain with positive actions, such as physical activity; (4) Encounter the unhappy thing will vent anger on others and often lose his temper. The responses to these questions were assigned the following scores: 1 (not at all), 2 (several days), 3 (more than half the day), 4 (nearly every day) (30). Cronbach's α was 0.74.

Mental health was measured using the patient health questionnaire (PHQ), the generalized anxiety questionnaire (GAD) and calculations of scores were based on a previous study (38, 39). The total stress subscale score was divided into normal (0–4), mild stress (5–9), moderate stress (10–14), and severe stress (15–21); the PHQ-9 scale was divided into normal (0–4), mild (5–9), moderate (10–14), moderate severe (15–19) and severe (20-27) (30, 40).

### Statistical analysis

Both in two rounds of survey, descriptive statistics were calculated for sociodemographic characteristics, lifestyles health behaviors and mental health. The chi-square test was used to analyze the prevalence of depression and anxiety in general data and health behaviors. The scores of the PHQ-9 and GAD-7 scales were expressed as multivariate variables in chi-square test and dichotomous variable in logistic regression. We also build a multiple lifestyle health behavior index to measure overall behaviors during the pandemic based on previous study (unpublished data). The ST was scored as 1 (> 4 h/d), 2 (2–4 h/d), or 3 (≤ 2 h/d). The PA was scored as 1 (<3 days) or 2 (≥3 days). The SSBs were scored as 5 (none), 4 (< 1 bottle per day), 3 (one bottle per day), 2 (two to three bottles per day), and 1 (more than four bottles per day). The frequency of diet was scored as 3 (increased), 2 (unchanged), and 1 (decreased). Health foods, including the Chinese herbal medicine vitamin, were scored as 1 (yes) and 2 (no). Worse appetite and vigor than before were scored as 1 (none), 2 (sometimes), 3 (half of the day), and 4 (all the time). The multiple lifestyle health behavior index was calculated as the sum of these behavior scores. In terms of coping styles, items 1 and 3 were reverse scoring items, and items 2 and 4 were forward scoring items. Then, the total score of the entries was calculated. We used linear regressions to calculate the association between sociodemographic characteristics, lifestyle behaviors, and mental health, with a significance level of *P* < 0.05.

The chi-square test was used for comparison between the two rounds of survey. The associations are presented in the form of regression coefficients and their 95% confidence intervals (CIs). Statistical analysis was performed using SPSS Statistics 23.0.

## Results

In the second round of surveys, there were 15 936 college students, 6 690 males (42.0%), and 9 246 females (58.0%), with a mean age of 20.58 ± 1.81 years. The prevalence rates of depressive symptoms and anxiety symptoms during the COVID-19 pandemic were 30.7 and 23.9%, respectively. The daily ST > 4 h/d, 2–4 h/d and ≤ 2 h/d of college students were 28.4, 37.1, 34.5% and the PA < 3 d/w and PA ≥ 3 d/w accounted for 54.6 and 45.4%; 35.4% of the college students had sugar sweetened beverages intake, 6.2 and 20.4% of the college students had Chinese herbal medicine and vitamin intake, 19.0 and 16.1% of the college students had reported worse sleep and diet rhythmicity than previous conditions, respectively. Similar results of the second survey are shown in Tables 1, 2. There were also some significant results between different school areas and health behaviors in Table 3.

**Table 1:** The prevalence between health behaviors and depression among college students.

**Demographic** **variables**	**Total**	**Depression**				***χ^2^*** **value**
		**Mild** **stress**	**Moderate** **stress**	**Moderate–severe** **stress**	**Severe** **stress**	
**Gender**						69.37^**^
Male	6,690	1,177 (17.6)	453 (6.8)	293 (4.4)	89 (1.3)	
Female	9,246	1,959 (21.2)	577 (6.2)	253 (2.7)	73 (0.8)	
**Age (years)**						25.83^**^
≤ 19	4,978	907 (18.2)	287 (5.8)	142 (2.9)	42 (0.8)	
20–22	9,097	1,852 (20.4)	596 (6.6)	336 (3.7)	100 (1.1)	
≥23	1,861	377 (20.3)	147 (7.9)	68 (3.7)	20 (1.1)	
**Regional areas**						31.68^**^
Area 1	3,608	762 (21.1)	257 (7.1)	136 (3.8)	37 (1.0)	
Area 2	6,336	1,248 (19.7)	400 (6.3)	196 (3.1)	44 (0.7)	
Area 3	1,487	270 (18.2)	88 (5.9)	52 (3.5)	18 (1.2)	
Area 4	4,505	856 (19.0)	285 (6.3)	162 (3.6)	63 (1.4)	
**Students type**						41.17^**^
Medical students	8,883	1,727 (19.4)	535 (6.0)	255 (2.9)	70 (0.8)	
Non-medical students	7,053	1,409 (20.0)	495 (7.0)	291 (4.1)	92 (1.3)	
**Grade**						55.15^**^
Freshmen	4,406	789 (17.9)	267 (6.1)	136 (3.1)	36 (0.8)	
Sophomore	3,923	740 (18.9)	234 (6.0)	137 (3.5)	34 (0.9)	
Junior	3,740	829 (22.2)	244 (6.5)	120 (3.2)	50 (1.3)	
Senior	2,671	530 (19.8)	190 (7.1)	103 (3.9)	33 (0.8)	
Fifth-grade	1196	248 (20.7)	95 (7.9)	50 (4.2)	9 (0.8)	
**ST**						293.41^**^
>4 h	4,532	1,024 (22.6)	425 (9.4)	213 (4.7)	90 (2.0)	
2–4 h	5,920	1,193 (20.2)	308 (5.2)	136 (2.3)	28 (0.5)	
≤ 2 h	5,484	919 (16.8)	297 (5.4)	197 (3.6)	44 (0.8)	
**PA**						121.17^**^
≥3 d	7,237	1280 (17.7)	371 (5.1)	192 (2.7)	67 (0.9)	
< 3 d	8,699	1,856 (21.3)	659 (7.6)	354 (4.1)	95 (1.1)	
**Sugar beverages**						159.88^**^
None	1,0287	1,898 (18.5)	598 (5.8)	276 (2.7)	87 (0.8)	
One to two bottle	4,510	967 (21.4)	344 (7.6)	206 (4.6)	50 (1.1)	
Three to four bottles	707	164 (23.2)	56 (7.9)	39 (5.5)	10 (1.4)	
More than five bottles	432	107 (24.8)	32 (7.4)	25 (5.8)	15 (3.5)	
**Vitamin**						3.92
Yes	3,255	660 (20.3)	226 (6.9)	115 (3.5)	37 (1.1)	
No	12,681	2,476 (19.5)	804 (6.3)	431 (3.4)	125 (1.0)	
**Chinese herbal medicine**						66.22^**^
Yes	995	235 (23.6)	91 (9.1)	58 (5.8)	21 (2.1)	
No	14,941	2,901 (19.4)	939 (6.3)	488 (3.3)	141 (0.9)	
**Worse appetite than before**						10296.46^**^
None	13,504	1,990 (14.7)	517 (3.8)	155 (1.1)	48 (0.4)	
Sometimes	1,788	1,067 (59.7)	332 (18.6)	123 (6.9)	22 (1.2)	
Half of the day	520	70 (13.5)	154 (29.6)	247 (47.5)	32 (6.2)	
All the time	124	9 (7.3)	27 (21.8)	21 (16.9)	60 (48.4)	
**Worse vigor than before**						14967.08^**^
None	11,647	1,069 (9.2)	190 (1.6)	42 (0.4)	8 (0.1)	
Sometimes	3,185	1,873 (58.8)	511 (16.0)	112 (3.5)	16 (0.5)	
Half of the day	883	168 (19.0)	291 (33.0)	339 (38.4)	46 (5.2)	
All the time	221	26 (11.8)	38 (17.2)	53 (24.0)	92 (41.6)	
**Frequency of diet**						715.49^**^
Decreased	2,558	597 (23.3)	275 (10.8)	146 (5.7)	40 (1.6)	
Increased	2,548	638 (25.0)	272 (10.7)	171 (6.7)	62 (2.4)	
Unchanged	10,830	1,901 (17.6)	483 (4.5)	229 (2.1)	60 (0.6)	
**Survey time**						81.28^**^
First survey	11,787	2,022 (17.2)	569 (4.8)	342 (2.9)	120 (1.0)	
Second survey	15,936	3,136 (19.8)	1,030 (6.5)	546 (3.4)	162 (1.0)	

** P < 0.01.

**Table 2:** The prevalence between health behaviors and anxiety among college students.

**Demographic variables**	**Total**	**Anxiety**			***χ^2^*** **value**
		**Mild stress**	**Moderate stress**	**Severe stress**	
**Gender**					50.95^**^
Male	6,690	1,076 (16.1)	461 (6.9)	92 (1.4)	
Female	9,246	1,649 (17.8)	409 (4.4)	117 (1.3)	
**Age (years)**					52.30^**^
≤ 19	4,978	749 (15.0)	218 (4.4)	50 (1.0)	
20–22	9,097	1,649 (18.1)	535 (5.9)	129 (1.4)	
≥23	1,861	330 (17.7)	117 (6.3)	30 (1.6)	
**Regional areas**					57.08^**^
Area 1	3,608	696 (19.3)	213 (5.9)	56 (1.6)	
Area 2	6,336	1,023 (16.1)	312 (4.9)	52 (0.8)	
Area 3	1,487	224 (15.1)	68 (4.6)	25 (1.7)	
Area 4	4,505	782 (17.4)	277 (6.1)	76 (1.7)	
**Students type**					75.07^**^
Medical students	8,883	1,427 (16.1)	407 (4.6)	84 (0.9)	
Nonmedical students	7,053	1,298 (18.4)	463 (6.6)	125 (1.8)	
**Grade**					63.31^**^
Freshmen	4,406	638 (14.5)	209 (4.7)	42 (1.0)	
Sophomore	3,923	671 (17.1)	208 (5.3)	42 (1.1)	
Junior	3,740	692 (18.5)	225 (6.0)	65 (1.7)	
Senior	2,671	499 (18.7)	157 (5.9)	45 (1.7)	
Fifth-grade	1,196	225 (18.8)	71 (5.9)	15 (1.3)	
**ST**					154.40^**^
>4 h	4532	884 (19.5)	309 (6.8)	111 (2.4)	
2–4 h	5,920	1007 (17.0)	231 (3.9)	48 (0.8)	
≤ 2 h	5,484	834 (15.2)	330 (6.0)	50 (0.9)	
**PA**					62.88^**^
≥3 d	7,237	1095 (15.1)	337 (4.7)	84 (1.2)	
< 3 d	8,699	1630 (18.7)	533 (6.1)	125 (1.4)	
**Soda beverages**					166.56^**^
None	10,287	1,582 (15.4)	460 (4.5)	114 (1.1)	
One bottle	4,510	909 (20.2)	313 (6.9)	65 (1.4)	
One to two bottle	707	135 (19.1)	55 (7.8)	16 (2.3)	
Three to four bottles	432	99 (22.9)	42 (9.7)	14 (3.2)	
More than five bottles					
**Vitamin**					67.36^**^
Yes	995	208 (20.9)	89 (8.9)	30 (3.0)	
No	14,941	2,517 (16.8)	781 (5.2)	179 (1.2)	
**Chinese herbal medicine**					4.62
Yes	3,255	584 (17.9)	190 (5.8)	48 (1.5)	
No	12,681	2,141 (16.9)	680 (5.4)	161 (1.3)	
**Worse appetite than before**					11881.55^**^
None	13,504	1,405 (10.4)	266 (2.0)	62 (0.5)	
Sometimes	1,788	1,210 (67.7)	223 (12.5)	37 (2.1)	
Half of the day	520	92 (17.7)	357 (68.7)	40 (7.7)	
All the time	124	18 (14.5)	24 (19.4)	70 (56.5)	
**Worse vigor than before**					14770.26^**^
None	11,647	620 (5.3)	67 (0.6)	13 (0.1)	
Sometimes	3,185	1,849 (58.1)	253 (7.9)	28 (0.9)	
Half of the day	883	213 (24.1)	506 (57.3)	65 (7.4)	
All the time	221	43 (19.5)	44 (19.9)	103 (46.6)	
**Frequency of diet**					638.70^**^
Decreased	2,558	588 (23.0)	212 (8.3)	56 (2.2)	
Increased	2,548	595 (23.4)	285 (11.2)	68 (2.7)	
Unchanged	10,830	1542 (14.2)	373 (3.4)	85 (0.8)	
**Survey time**					163.92^**^
First survey	11,787	1,465 (12.4)	462 (3.9)	171 (1.5)	
Second survey	15,936	2,725 (17.1)	870 (5.5)	209 (1.3)	

** P < 0.01.

**Table 3:** The difference regional areas of health behaviors among college students.

**Different behavior variables**	**Regional areas**				
	**Area 1**	**Area 2**	**Area 3**	**Area 4**	***P*** **-value**
**ST**					>0.05
≤ 2 h (low)	742 (13.5)	1917 (35.0)	286 (5.2)	2,539 (46.3)	
2–4 h (medium)	853 (14.4)	2167 (36.6)	281 (4.7)	2,619 (44.2)	
>4 h (high)	663 (14.6)	1622 (35.8)	242 (5.3)	2,005 (44.2)	
**PA**					>0.05
≥3 d (high)	1,014 (14.0)	2586 (35.7)	389 (5.4)	3,248 (44.9)	
< 3 d (low)	1,244 (14.3)	3120 (35.9)	420 (4.8)	3,915 (45.0)	
**SSBs**					**< 0.01**
None	1,369 (13.3)	3,965 (38.5)	462 (4.5)	4,491 (43.7)	
One to two bottle	695 (15.4)	1,441 (32.0)	271 (6.0)	2,103 (46.6)	
Three to four bottles	116 (16.4)	188 (26.6)	49 (6.9)	354 (50.1)	
More than five bottles	78 (18.1)	112 (25.9)	27 (6.3)	215 (49.8)	
**Chinese herbal medicine**					**< 0.01**
No	2,091 (14.0)	5,442 (36.4)	745 (5.0)	6,663 (44.6)	
Yes	167 (16.8)	264 (26.5)	64 (6.4)	500 (50.6)	
**Vitamin**					**< 0.01**
No	1,710 (13.5)	4686 (37.0)	631 (5.0)	5,654 (44.6)	
Yes	548 (16.8)	1020 (31.3)	178 (5.5)	1,509 (46.4)	
**Worse appetite than before**					**< 0.01**
None	1,863 (13.8)	4912 (36.4)	681 (5.0)	6,048 (44.8)	
Sometimes	297 (16.6)	610 (34.1)	92 (5.1)	789 (44.1)	
Half of the day	78 (15.0)	152 (29.2)	27 (5.2)	263 (50.6)	
All the time	20 (16.1)	32 (25.8)	9 (7.3)	63 (50.8)	
**Worse vigor than before**					>0.05
None	1,620 (31.9)	4,180 (35.9)	605 (5.2)	5,242 (45.0)	
Sometimes	462 (14.5)	1,177 (37.0)	42 (4.8)	426 (48.2)	
Half of the day	142 (16.1)	273 (30.9)	42 (4.8)	426 (48.2)	
All the time	34 (15.4)	76 (34.4)	13 (5.9)	98 (44.3)	
**Meal frequency**					**< 0.01**
Decreased	385 (15.1)	824 (32.3)	146 (5.7)	1,193 (46.8)	
Increased	349 (13.6)	919 (35.9)	130 (5.1)	1,160 (45.3)	
Unchanged	1,524 (14.1)	3,963 (36.6)	533 (4.9)	1,193 (44.4)	

The bold values indicates the significant results.

The comparison of health behaviors at the different survey times is shown in Table 4. In the first survey, ST > 4 h was higher than in the second survey (55.1 vs. 44.9%). The other results were also shown in PA (≥ 3 d, first vs. second = 32.3 vs. 67.7%), decreased diet rhythmicity (changed, first vs. second = 35.0 vs. 65.0%), Chinese herbal medicine (have, first vs. second = 56.9 vs. 43.1%) and vitamin (have, first vs. second = 41.4 vs. 58.6%), worse vigor than before (all the time, first vs. second = 46.6 vs. 53.4%) (all *P* < 0.01). There were significant differences between the two surveys and different levels of mental health. Compared with the first survey, the prevalence of depression and anxiety in the second rounds of the survey was higher (depression: first vs. second = 25.9 vs. 30.6%; anxiety: first vs. second = 17.8 vs. 23.9%).

**Table 4:** The association between health behaviors and mental health among college students.

**Different behavior variables**	**Survey time**		***χ^2^*** **value**
	**First survey**	**Second survey**	
**ST**			1125.34^**^
>4 h (high)	5,570 (55.1)	4,532 (44.9)	
2–4 h (medium)	3,706 (38.5)	5,920 (61.5)	
≤ 2 h (low)	2,511 (31.4)	5,483 (68.6)	
**PA**			742.65^**^
< 3 d (low)	8,334 (48.9)	8,699 (51.1)	
≥3 d (high)	3,453 (32.3)	7,236 (67.7)	
**Chinese herbal medicine**			212.08^**^
No	1,312 (56.9)	995 (43.1)	
Yes	10,475 (41.2)	14940 (58.8)	
**Vitamin**			3.40
No	2,302 (41.4)	3,255 (58.6)	
Yes	9,485 (42.8)	12,680 (57.2)	
**Worse appetite than before**			82.90^**^
None	10,349 (43.4)	13,503 (56.6)	
Sometimes	1,007 (36.0)	1,788 (64.0)	
Half of the day	290 (35.8)	520 (64.2)	
All the time	141 (53.2)	124 (46.8)	
**Worse vigor than before**			204.49^**^
None	9,428 (44.7)	11,647 (55.3)	
Sometimes	1,752 (35.5)	3,184 (64.5)	
Half of the day	414 (31.9)	883 (68.1)	
All the time	193 (46.6)	221 (53.4)	
**Frequency of diet**			294.82^**^
Increased	1,591 (38.3)	2,558 (61.7)	
Decreased	1,157 (31.2)	2,548 (68.8)	
Unchanged	9,039 (45.5)	10,829 (54.5)	

** P < 0.01.

For further observation, we have attached the results of the first survey, the first survey results of the prevalence between health behaviors and depression, anxiety symptoms were shown in **previous data** (41).

In logistic regression analysis, the correlation of depression and anxiety in the second rounds of the survey was higher than that in the first survey (depression: *OR* = 1.26, 95% *CI* = 1.16, 1.36; anxiety*: OR* = 1.47, 95% *C*I = 1.35, 1.60). These results are shown in Table 5. After adjusting for confounding factors, ST > 4 h/d was positively correlated with depression (*OR* = 1.72, *95% CI*: 1.58,1.87) and anxiety symptoms (*OR* = 1.39, *95% CI*: 1.27,1.53) compared with daily ST ≤ 2 h/d. Daily 2–4 h/d ST was also positively correlated with depression (*OR* = 1.09, *95% CI*: 1.001,1.18), but not anxiety symptoms (*OR* = 0.98, *95% CI*: 0.90,1.08) compared with daily ST ≤ 2 h/d. After adjusting for confounding factors, compared with PA ≥ 3 d/w, < 3 d/w was positively correlated with depression (*OR* = 1.43, *95% CI*: 1.33,1.53) and anxiety symptoms (*OR* = 1.34, *95% CI*: 1.31–1.63). College students who reported higher SSB intake, Chinese herbal medicine and vitamin consumption, and changed frequency of diet had higher depression and anxiety symptoms. Moreover, the scores on the second survey were higher than those on the first survey for anxiety and depression symptoms.

**Table 5:** The association between health behaviors and mental health among college students.

**Different behavior variables**	**Anxiety and depression**	
	**Anxiety *OR* (95% *CI*)**	**Depression *OR* (95% *CI*)**
**ST**		
≤ 2 h (low)	1.00	1.00
2–4 h (medium)	0.98 (0.90,1.08)	1.09 (1.001,1.18)^*^
>4 h (high)	1.39 (1.27,1.53)^**^	1.72 (1.58,1.87)^**^
**PA**		
≥3 d (high)	1.00	1.00
< 3 d (low)	1.34 (1.24,1.44)^**^	1.43 (1.33,1.53)^**^
**Sugar beverages**		
None	1.00	1.00
One to two bottle	1.52 (1.40,1.65)^**^	1.40 (1.30,1.51)^**^
Three to four bottles	1.52 (1.28,1.80)^**^	1.59 (1.36,1.86)^**^
More than five bottles	2.06 (1.68,2.53)^**^	1.82 (1.49,2.22)^**^
**Chinese herbal medicine**		
No	1.00	1.00
Yes	1.60 (1.39,1.84)^**^	1.62 (1.23,1.39)^**^
**Vitamin**		
No	1.00	1.00
Yes	1.09 (1.0,1.20)	1.09 (1.00,1.18)
**Worse appetite than before**		
None	1.00	1.00
Sometimes	31.92 (28.67,35.53)^**^	29.48 (26.59,32.68)^**^
Half of the day	120.86 (96.65,151.14)^**^	167.59 (120.90,232.31)^**^
All the time	94.02 (63.69,138.79)^**^	133.40 (74.30,239.53)^**^
**Worse vigor than before**		
None	1.00	1.00
Sometimes	31.90 (27.93,36.43)^**^	25.44 (22.07,29.33)^**^
Half of the day	109.21 (75.58,157.81)^**^	119.97 (73.80,195.04)^**^
All the time	62.89 (34.52,114.58)^**^	65.83 (30.63,141.48)^**^
**Frequency of diet**		
Decreased	1.00	1.00
Increased	1.17 (1.04,1.32)	1.15 (1.03,1.29)^*^
Unchanged	0.45 (0.41,0.50)	0.47 (0.43,0.51)^**^
Survey time		
First survey	1.0	1.0
Second survey	1.47 (1.35,1.60)^**^	1.26 (1.16,1.36)^**^

Adjusted for age, grade, gender, student types, regional areas, residential areas.

* P < 0.05,

** P < 0.01.

For further observation, we have attached the results of the first survey, the first survey results of the association between health behaviors and mental health among college students were shown in **previous data**.

In Tables 6, 7 mediation and moderate analyses were conducted to determine whether relationships between lifestyle health behaviors and mental health symptoms were (to some extent) mediated by coping responses to COVID-19. These results illustrate that there were significant indirect effects (of small magnitude) alongside significant direct effects (of moderate-to-large magnitude) for lifestyle health behaviors. Compared with males, females attenuated the higher coping style effect and the effect of a better lifestyle on anxiety and depression.

**Table 6:** Model characteristics for the conditional process analysis.

	**Anxiety**		**Depression**	
	**B**	***P*** **–value**		***P*** **–value**
Lifestyle behaviors	−1.43	**< 0.001**	−1.78	**< 0.001**
Coping style	−0.25	**< 0.001**	−0.32	**< 0.001**
Gender	−4.62	**< 0.001**	−5.22	**< 0.001**
Lifestyle behaviors x gender	0.24	**< 0.001**	0.29	**< 0.001**
Gender x Coping style	−0.31	>0.05	−0.07	>0.05
R^2^	0.26		0.27	
F	1137.64		1185.98	

Mediate variables, coping style; Moderated variables, gender; Independent variables, lifestyle health behaviors; Dependent variables, mental health symptoms. The bold values indicates the significant results. x, interaction effect.

**Table 7:** Bootstrapped conditional direct and indirect effects.

			**Anxiety**			**Depression**		
			**Effect**	**SE**	**(LL,UL)**	**Effect**	**SE**	**(LL,UL)**
Direct effect							
	Predictor	Lifestyle health behaviors						
	Moderator	Male	−1.1883	0.0219	−1.2312, −1.1453	−1.50	0.0274	−1.55, −1.44
		Female	−0.9443	0.0205	−0.9846, −0.9041	−1.21	0.0257	−1.26, −1.16
Indirect effect								
	Predictor	Coping style						
	Moderator	Male	−0.0243	0.0039	−0.0332, −0.0179	−0.0329	0.005	−0.0431, −0.0235
		Female	−0.0390	0.0047	−0.0491, −0.0306	−0.0559	0.0059	−0.0685, −0.0449

Mediate variables, coping style; Moderated variables, sex; Independent variables, lifestyle health behaviors; Dependent variables, mental health symptoms. The model was controlled for age, grade, student types, regional areas, residential areas.

## Discussion

### Principal findings

Based on the previous study, the second round of national survey was conducted to investigate the association between lifestyle behavior changes, coping styles, COVID-19-related information, and mental health symptoms in Chinese college students at the beginning of the pandemic and 3 months later. It is helpful to provide a good theoretical basis for subsequent intervention. Our nationwide two rounds of survey had some main findings.

**Firstly**, in the first survey, only 21.3% had ST < 2 h, nearly 50.0% of the college students ST were more than 4 h per day, and 70.7% of the college students had inadequate activity (PA < 3 d/w) at the beginning of the pandemic (30). Some researchers have shown that during the pandemic, ST was higher in young adults (16, 42). These results also showed that the pandemic had different effects on people of different ages. The ST results of college students in this study were higher than those in previous studies (31). While most previous studies have investigated ST exposure in all age groups, our study focused on college students, who are more prone to “infodemic” due to the freedom of time and new age trends (43). **Secondly**, at the same time, we found that the PA deficiency of adolescents was nearly 71% (30), higher than that of adults during the pandemic period (nearly 60%) (16), and the prevalence of insufficient PA of college students was higher than the previous research level (51%) (44) in our study. A possible reason was that our study involved different affected areas in the country, and some areas were in cities with a high incidence of pandemics, so physical inactivity was high. After 3 months, in the second survey, the prevalence of ST (>4 h/d) and PA (<3 d/w) were 28.4 and 54.6%, respectively. This downward trend shows that people can learn to change bad behavior as the pandemic occurs, along with the availability of information and effective implementation of policy intervention. **Thirdly**, 13.7 and 15.6% of the college students had soda and tea beverage intake, 11.1 and 19.5% of the college students had Chinese herbal medicine and vitamin intake, and 15.6% of the college students reported decreased diet rhythmicity. In the second survey, the prevalence of SSB intake, Chinese herbal medicine and vitamin intake, and decreased diet rhythmicity were 35.4, 6.2, 11.7, and 16.0%, respectively. **Fourthly**, in our study, 25.9 and 17.8% of college students reported depression and anxiety symptoms, respectively. In the second survey, the prevalence rates of depression and anxiety were 30.6 and 23.9%, respectively. **Finally** and interestingly, both in two rounds of survey, we also found that the individual and multiple lifestyle health behavior scores were associated with depression and anxiety symptoms among college students (the β value of depression and anxiety in second survey were 1.38 and 1.08, P all<0.01, adjusted model). Moreover, we found that coping style with COVID-19 partially mediated the associations between some of the related lifestyle behaviors and anxiety and depression. The results of the conditioned process model analysis support our previous hypothesis that lifestyle, health behaviors, and coping styles are predictors of anxiety and depressive symptoms, and their direct and indirect effects on outcomes are moderated at the gender level. This is our large-scale population-based two rounds of study investigating lifestyle behaviors, COVID-19-related social stressors, and psychological effects among college students in China during the first pandemic to 3 months.

### Comparison with other studies

Such strict social and economic measures are expected to have important effects on people's health and lives (45), thus it also causes a variety of psychological problems, such as bad-tempered, anxiety and depression (21, 22). In this study, depressive symptoms of college students were higher than in the general survey before pandemic (46, 47) and another cross-sectional epidemiological study during pandemic (48). In addition, the depressive and anxiety symptoms of second survey were higher than first survey. Another study reported that a high prevalence of intangible psychological stress caused by ST was positively associated with depressive symptoms in the case of longer isolation periods (49). These results suggest that the psychological impact of the pandemic should not be ignored, as in previous studies adolescents have been shown to have significantly higher rates of post-traumatic stress disorder (PTSD) symptoms as a result of experiencing isolation (50). Moreover, similar results have been obtained in animal studies, when rodent adolescent isolation occurs chronically, over 1 week or longer, it has even more profound effects (51). In addition, according to two rounds of survey, more attention should be given to the behavior and symptoms of depression and anxiety among women (9, 52), non-medical backgrounds and college students in high risk areas. Furthermore, women are more likely to exhibit unhealthy behaviors and symptoms of depression and anxiety than men based on two rounds of survey, perhaps because women are more emotional and emotional and are relatively prone to stress (30). The incidence of depressive symptoms is reported to be higher than that in men among previous studies (16, 21, 53, 54). According to the second surveys, we found the prevalence of depressive symptoms and anxiety symptoms during COVID-19 was 30.7 and 23.9%, respectively, which were all higher than first survey, this shows that at the beginning of the pandemic, people's panic and uncertainty about the virus caused some psychological problems, and as the number of confirmed cases gradually increased, it reached a peak, so the psychological problems became more serious.

Some research articles have also found that whether prior to or during the pandemic pandemic, high levels of ST (>4 h) was associated with anxiety and depression, therefore, our research has carried out two rounds of survey, aims to observe whether there is this kind of phenomenon during pandemics, the results also confirmed that high levels of ST during the pandemic caused psychological problems; and originally found in young people's survey, based on sedentary ST are also an important factor in assessing health behavior (30, 55, 56). During COVID-19, we received the amount of information through social media (screen time) has increased dramatically, but the quality control, and pieces of information, some unfiltered information coming into our view through social media, coupled with the uncertainty about COVID-19, is why its negative emotions are also increasing (57–59). The fragmentation of information in news can only lead to unnecessary trouble and fear. There is growing evidence that exposure to traumatic or threatening content from ST can influence fear conditioning by activating fear circuits in the brain and may produce symptoms of post-traumatic stress disorder, particularly flashbacks (59). But with the development of the pandemic, the news about this part have been confirmed, so everyone's ST will gradually decline, and then transferred to other activities, such as the increase of physical activity; however, due to the uncertainty and uncertainty of COVID-19 itself, it will bring bad emotional aftereffects to people (57, 60), that is why the prevalence of mental health problems continued to increase in the second study.

Interestingly, we also found that medium-level ST was negatively associated with college students' depression and anxiety symptoms in first survey (30) but not in second survey. One possible reason is that in the early stage of COVID-19 pandemic, people paid more attention to relevant news and information, including patient information on diagnosis and action and pandemic prevention measures. These social media can provide online information, such as Health, Peace and Good Doctor, Ali Dingxiang BBS, wechat), As well as online office and school routines (i.e., online classes, online work, exercise programs on electronic devices), the time spent on ST is basically useful (42), and suggested a U-shaped non-linear association between ST and depression symptoms in previous study (61). Non-linear associations between sitting time and major depressive episode onset might explain this inconsistency (62). It does take time for people to explore information about COVID-19, make sense of the messages they receive and share them with those close to them through social media, and it is possible that appropriate information seeking behavior may reduce mental health due to uncertainty during the COVID-19 pandemic (30, 63), many studies have also shown that, to some extent, social media, such as direct communication (i.e., messaging, video chatting, etc.), has been shown to improve healthy mental states (64); However, given the “information pandemic” and emotional contagion through online social networks, spending excessive time searching for COVID-19 news on ST is debatable (42). Our results also support these theories and current phenomena; not necessarily, the more screen time, the worse but also have a critical value. With the development of the pandemic, people's living habits have returned to normal, and they have more or less understanding of COVID-19 news. The higher the video time itself, the worse the psychological problems, so that explains why the results after 3 months were the opposite of the original results, but similar to those of the previous study. At the physical activity level, there has been increasing evidence, both during and before epidemics, that high PA is associated with many health benefits (65, 66) and had a positive impact during COVID-19 (30, 67). It is also fully confirmed that PA can affect the mental health of college students (68, 69), while low PA levels lead to more depression, which is basically consistent with previous studies (30, 31). Outcomes were similar between PA and depressive symptoms during the COVID-19 pandemic, and in addition, people's behavior was limited during the pandemic, which could lead to adverse psychological outcomes (70). Similar results were also shown in second survey. This also suggests that both at the beginning of the pandemic and three months later, together with pre-pandemic studies, suggest that the association between physical activity levels and mental health is relatively clear, meaning that higher levels of physical activity are associated with better mental health. Di Renzo et al. reported that a slight increased physical activity, which was similar with our results (71). Although the second screen time and physical activity results were lower than the first prevalence, the psychological problems still increased, indicating that the factors affecting psychological problems include but are not limited to these two behaviors, prompting us to further consider whether other factors play a role.

Therefore in our study, we also explored SSBs (including soda and tea beverage in first survey, whereas only SSBs in second survey) intake. According to our knowledge, SSBs themselves cause mental health problems in different age groups (72, 73). Some studies concerned the correlation between SSBs and physical health— a higher intake of SSBs had a positive effect on infection rates by COVID-19, and the same results also demonstrated that the intake of fruits had a positive effect on mortality by COVID-19 (20), few study pay attention to the mental health and SSBs. The reason for our focus on sugar-sweetened beverage consumption during the COVID-19 outbreak is to promote the development of healthy behaviors and reduce the further adverse psychological effects of unhealthy behaviors among adults, especially new age youth, who are mainly college students, during COVID-19. Because COVID-19 itself can cause psychological problems for a long time. In a large environment such as COVID-19, people will increase and decrease their intake of SSBs when informed by media information, as students' lifestyles change, their thoughts also change, and SSB intake causes mental health problems not only by themselves but also by a range of behaviors, also known as the so called “Theory of Planned Behavior (TPB)” (74), this included ST and PA, which were mentioned earlier, they influence each other. Food intake was reportedly increased because of emotional eating as a means of comfort and to feel better in response to anxious states (71, 75), with higher levels of this reported behavior in women (71). Moreover, because of the infodemic, students also take the action of buying Chinese herbal medicine (e.g., radix isatidis and Yunnan Baiyao) and vitamins to improve their physical fitness to further increase unnecessary panic. Whereas, in second survey, the phenomenon has declined slightly. Possible reason was that with the time of the epidemic, people's lives have gradually returned to normal even if they are still in quarantine, and the news about COVID-19 is slowly seeping into their brains. People have also learned how to protect themselves through practice and the news media, so the related hoarding behavior has decreased. Sleep was one of the most crucial factors to recover whenever someone suffered diseases or lifestyle events; otherwise, sleep disturbances would cause anxiety and panic problems during the COVID-19 pandemic (76, 77).

In addition, we also explored the students' degree of diet rhythmicity; compared to decreased students who reported unchanged and increased, they had better mental health problems, which suggested that in situations such as COVID-19, proper timing of diet habits may be necessary to increase unnecessary panic attacks and negative symptoms (78). We also compared two rounds of survey, ST > 4 h, changed decreased diet rhythmicity, Chinese herbal medicine and vitamin intake, the frequency of all the time worse vigor than before were higher than in the second survey.

In order to better understand the impact of comprehensive behaviors on mental health, we attempted to use multiple health indicators, which are designed to explore the possible combined effects of behaviors both in two rounds of surveys. We all know that among students who engage in one healthy behavior, clusters of healthy behaviors are also more likely to engage in other healthy behaviors. In our study, the index included appetite, vitality, diet rhythm, SSBs, ST, and PA (79). To get a fuller picture of the impact of behavior on psychological problems, because coping style is a key factor affecting psychological problems, which has also been reported in previous studies (80), we included coping styles as a variable, the current study revealed a significant moderate effect of coping style on the development of anxiety and depression symptoms in college students among two rounds of survey. Coping style can effectively decrease all indices of distress and is positively associated with well-being (81, 82), and more use of cognitive and prosocial coping behaviors was associated with fewer mental health problems (81), during COVID-19, this research identified positive and negative correlations between presented coping styles and manifested psychopathology (82). However, there is no study explored the potential association. Based on these, the results of the conditional process model analysis supported our hypotheses that lifestyle health behaviors and coping style were both predictors for anxiety and depression symptoms, and their direct and indirect effects were all moderated by the level of gender. Although we have not compared the trend between two rounds of survey, our study further proved the robustness of the model through two rounds of survey.

We also concerned the influence of COVID-19 perception in previous study (21), nearly 94.7% of the students paid close attention to whether they had symptoms such as fever, cough, sneezing or fatigue. In the first survey, logistic regression association in the study showed that perceived physical symptoms, such as fever and cough, were significantly associated with higher anxiety and depression symptoms. A previous study also showed that physical symptoms can have a significant impact on psychological responses (21, 83). The correlation between physical health perception and depression has also been demonstrated (84). In these two rounds of survey, family/friends living with students who had direct/indirect contact with COVID-19 affected areas were also significantly associated with increased anxiety levels. It can be concluded that because students care about the health of their families and friends. In addition, there was no clear treatment, so the corresponding increase in anxiety and depression symptoms (29). In second survey, we also examined the correlation between the duration of home isolation and mental health symptoms, interestingly, the shorter duration of home isolation were correlated with higher mental health symptoms. Although we have not compared the trend between two rounds of survey, our study further proved the robustness of the model through two rounds of survey.

### Limitations and strengths

Our study inevitably has some limitations. First, this study is a cross-sectional study that could not establish a causal relationship between lifestyle behaviors and mental health outcomes. Furthermore, the variables in this study were self-reported using an electronic questionnaire, and given the specific pandemic situation, we were unable to actually test college students, so there may be recall bias. Third, the biological mechanism underlying this association has not yet been directly assessed. Future longitudinal studies should further sample collection to explore possible psychosocial and biological pathways. Finally, we did not obtain the physical health status and objective lifestyle behaviors of the participants before pandemic in this study. We do not report the first study results, which have been reported in previous studies. Furthermore, our study is a continuous follow-up study conducted every 6 months to further clarify the relationship.

Our study results are also worthy of attention. First, we have a large sample of college students and different pandemic areas to compare regional differences in order to provide basic information to local schools and governments about the psychology and behavior of college students during home quarantine. Second, the timely grasp of the pandemic problem for domestic college students' psychology during the first pandemic, especially the basic situation of psychological and behavioral problems, can provide a reference; and we also investigated the second survey, further provide intervention measures for exploring the psychological and behavioral changes in different periods of the pandemic, and this survey has already investigated the third survey, changes in behavioral and psychological problems will be explored further. Third, during the period of isolation, on the one hand, people should not only reasonable and proper arrangement through the media spend time on news attention pandemic, the corresponding increase physical activity and reduce the occurrence of poor eating behaviors, such as reducing the intake of some health care products due to psychological problems, so as to further deepen the development of psychological problems, maintain a good pace of life, to further address possible follow-ups. Finally, on the other hand, the need to respond to the nationwide home quarantine measures has greatly restricted the movement of people, especially those who have been in contact with confirmed cases. Family isolation will have different degrees of psychological impact. This study can provide reference for subsequent research by asking relevant information. Therefore, this study took this as a starting point to carry out a nationwide survey of college students' mental health.

## Conclusion

Our study found that the psychological and behavioral results are different, with the second being higher than the first survey; adoption of healthy lifestyle behaviors and timely and correct understanding of COVID-19-related factors are negatively correlated with anxiety and depression symptoms. Relevant government departments should also focus on health education for college students with non-medical backgrounds to improve their understanding of the novel coronavirus. At the same time, the influence of the relevant government departments and universities should play an active and attract students to arrange appropriate activities during the period of popular, such as reducing recreational screen time, increased physical activity, maintaining a healthy diet, propaganda popular science knowledge and keep a good pace of life, especially in the family, during the period of separation further planning their lives and have a rest. Through two rounds of investigation and research, it is found that the psychological problems of college students should be continuously paid attention to and a series of intervention measures should be taken. This study provides some possible references from the behavioral style and living habits. Therefore, understanding the relationship between college students' lifestyle health behaviors and their psychology during home quarantine will help school leaders and the Ministry of Education urgently formulate and implement effective policies and interventions for college students' lifestyle health behaviors (85). We also tried to ask the two rounds of college students whether they consulted psychological support. Although the results of this study were not presented, they also provided a good investigation basis for this study.

## Data availability statement

The original contributions presented in the study are included in the article/Supplementary material, further inquiries can be directed to the corresponding author/s.

## Ethics statement

The studies involving human participants were reviewed and approved by Anhui Medical University. The patients/participants provided their written informed consent to participate in this study. Written informed consent was obtained from the individual(s) for the publication of any potentially identifiable images or data included in this article.

## Author contributions

YZ collected and arranged the data and drafted the manuscript. ST and XW were responsible for contacting cooperative units, participant recruitment, and data collection. YQ, XM, HG, ZZ, and PZ were all responsible for participant recruitment and data collection. FT obtained the funding and designed the study. All authors contributed to the article and approved the submitted version.
