# Supplementary material for: The correlation between lifestyle health behaviors, coping style, and mental health during the COVID-19 pandemic among college students: Two rounds of a web-based study
Source: Front Public Health. 2023 Jan 12;10:1031560. doi: 10.3389/fpubh.2022.1031560 (PMC9878348; doi:10.3389/fpubh.2022.1031560)
Supplement: Supplementary file 1 [file Data_Sheet_1.doc]

In the first stage, according to the geographic location and cooperation intention, 16 provinces or municipalities were selected: Wuhan city, the neighboring province of Hubei (Henan, Anhui, Jiangxi, Hunan, Chongqing and Shanxi), first-tier cities (Beijing, Shanghai and Guangzhou) and other provinces (Jiangsu, Guangxi, Yunnan, Xingjiang, Heilongjiang and Jilin). A total of 3-4 universities were randomly selected in Wuhan, Hubei, and 15 universities were randomly selected from other provinces or municipalities. In the second stage, 1 faculty member was randomly selected from each university, and 100-120 students from each grade (in general, 5 years for medical students and 4 years for nonmedical students) were invited to participate in an online survey although the Wenjuanxing platform (https://www.wjx.cn/). In total, 14,789 students were selected.


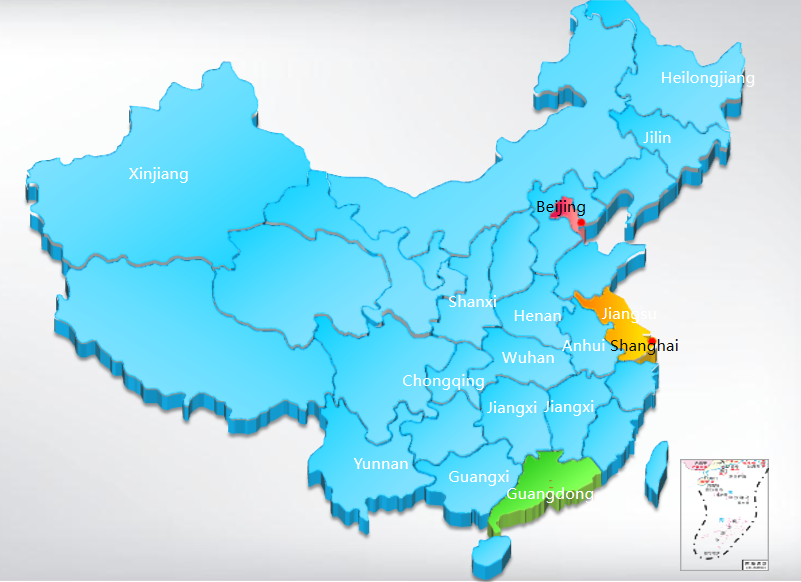


**Figure S1 Distribution of study sample**

**Supplemental file 1**

**Table S1 List of all the participants**

| **Location** | **Number of Universities** | **Faculties** | **Grade** | **Total** | **Valid sample** |
| --- | --- | --- | --- | --- | --- |
| Anhui | 1 | Medical×1 | Year 1-5 | 500 | 500 [100 per grade] |
| Heilongjiang | 1 | Non-medical×1 | Year 1-4 | 400 | 400 [100 per grade] |
| Guangdong | 1 | Medical×1 | Year 1-5 | 500 | 500 [100 per grade] |
| Guangxi | 1 | Non-medical×1 | Year 1-4 | 400 | 400 [100 per grade] |
| Jiangsu | 1 | Non-medical×1 | Year 1-4 | 400 | 400 [100 per grade] |
| Wuhan, Hubei [1] | 1 | Medical×1 | Year 1 | 135 | 111 |
|  |  | Medical×1 | Year 2 | 125 | 88 |
|  |  | Medical×1 | Year 3 | 138 | 66 |
|  |  | Medical×1 | Year 4 | 138 | 105 |
|  |  | Medical×1 | Year 5 | 146 | 146 |
| Jilin | 1 | Medical×1 | Year 1-5 | 500 | 500 [100 per grade] |
| Yunan | 1 | Medical×1 | Year 1-5 | 500 | 500 [100 per grade] |
| Shanghai | 1 | Non-medical×1 | Year 1-4 | 400 | 400 [100 per grade] |
| Jiangxi | 1 | Non-medical×1 | Year 1-4 | 400 | 400 [100 per grade] |
| Beijing | 1 | Medical×1 | Year 1 | 74 | 74 |
|  |  | Medical×1 | Year 2 | 76 | 69 |
|  |  | Medical×1 | Year 3 | 86 | 86 |
|  |  | Medical×1 | Year 4 | 79 | 79 |
|  |  | Medical×2 | Year 5 | 230 | 192 |
| Wuhan, Hubei [2] | 1 | Medical×1 / Non-medical×1 | Year 1 | 235 | 163 [Medical:76] |
|  |  | Medical×1 / Non-medical×1 | Year 2 | 330 | 131 [Medical:77] |
|  |  | Medical×1 / Non-medical×1 | Year 3 | 255 | 104 [Medical:40] |
|  |  | Medical×1 / Non-medical×1 | Year 4 | 327 | 102 [Medical:43] |
|  |  | Medical×1 / Non-medical×1 | Year 5 | 134 | 59 [Medical:58] |
| Wuhan, Hubei [3] | 1 | Medical×1 / Non-medical×8 | Year 1 | 1341 | 1044 [Medical:37] |
|  |  | Medical×1 / Non-medical×8 | Year 2 | 1400 | 859 [Medical:79] |
|  |  | Medical×1 / Non-medical×8 | Year 3 | 1378 | 938 [Medical:88] |
|  |  | Medical×1 / Non-medical×8 | Year 4 | 1281 | 568 [Medical:104] |
|  |  | Non-medical×1 | Year 5 | 0 | 3 |
| Xi’an | 1 | Non-medical×1 | Year 1 | 100 | 100 |
|  |  | Medical×1 | Year 2 | 100 | 100 |
|  |  | Non-medical×1 | Year 3 | 100 | 100 |
|  |  | Medical×1 / Non-medical×1 | Year 4 | 100 | 100 [Medical:52] |
|  |  | Medical×1 | Year 5 | 100 | 100 |
| Xinjiang | 1 | Medical×1 | Year 1-5 | 500 | 500 [100 per grade] |
| Henan | 1 | Medical×1 | Year 1-5 | 500 | 500 [100 per grade] |
| Hunan | 1 | Medical×1 | Year 1 | 146 | 135 |
|  |  |  | Year 2 | 82 | 57 |
|  |  |  | Year 3 | 73 | 70 |
|  |  |  | Year 4 | 79 | 70 |
|  |  |  | Year 5 | 78 | 68 |
| Wuhan, Hubei [4] | 1 | Non-medical×1 | Year 1-4 | 400 | 400 [100 per grade] |
| Chongqing | 1 | Medical×1 | Year 1 | 118 | 103 |
|  |  |  | Year 2 | 105 | 105 |
|  |  |  | Year 3 | 103 | 103 |
|  |  |  | Year 4 | 94 | 90 |
|  |  |  | Year 5 | 103 | 99 |
| **Total** | **19** |  |  | **14789** | **11787** |
